# Enhanced Heat Transfer for NePCM-Melting-Based Thermal Energy of Finned Heat Pipe

**DOI:** 10.3390/nano12010129

**Published:** 2021-12-31

**Authors:** Sameh E. Ahmed, Aissa Abderrahmane, Sorour Alotaibi, Obai Younis, Radwan A. Almasri, Wisam K. Hussam

**Affiliations:** 1 Department of Mathematics, Faculty of Science, King Khalid University, Abha 62529, Saudi Arabia; sehassan@kku.edu.sa; 2 Department of Mathematics, Faculty of Science, South Valley University, Qena 83523, Egypt; 3 Laboratoire de Physique Quantique de la Matière et Modélisation Mathématique (LPQ3M), University of Mascara, Mascara 29000, Algeria; a.aissa@univ-mascara.dz; 4 Mechanical Engineering Department, College of Engineering and Petroleum, Kuwait University, P.O. Box 5969, Safat 13060, Kuwait; 5 Department of Mechanical Engineering, College of Engineering at Wadi Addwaser, Prince Sattam Bin Abdulaziz University, Wadi Addwaser 11991, Saudi Arabia; oubeytaha@hotmail.com; 6 Department of Mechanical Engineering, Faculty of Engineering, University of Khartoum, Khartoum 11111, Sudan; 7 Department of Mechanical Engineering, College of Engineering, Qassim University, Buraydah 51452, Saudi Arabia; masri.radwan@qec.edu.sa; 8 School of Engineering, Australian College of Kuwait, Safat 12000, Kuwait; w.alsaadi@ack.edu.kw

**Keywords:** melting process, PCM, FEM, shell designs, tubes, branched fins, latent heat

## Abstract

Using phase change materials (PCMs) in energy storage systems provides various advantages such as energy storage at a nearly constant temperature and higher energy density. In this study, we aimed to conduct a numerical simulation for augmenting a PCM’s melting performance within multiple tubes, including branched fins. The suspension contained Al_2_O_3_/n-octadecane paraffin, and four cases were considered based on a number of heated fins. A numerical algorithm based on the finite element method (FEM) was applied to solve the dimensionless governing system. The average liquid fraction was computed over the considered flow area. The key parameters are the time parameter (100 ≤t≤600 s) and the nanoparticles’ volume fraction (0%≤φ≤8%). The major outcomes revealed that the flow structures, the irreversibility of the system, and the melting process can be controlled by increasing/decreasing number of the heated fins. Additionally, case four, in which eight heated fins were considered, produced the largest average liquid fraction values.

## 1. Introduction

In modern times, energy storage and conservation solutions are as important as ever due to depletion and climate change challenges. Phase change materials (PCMs) are at the forefront in this domain due to their low cost and availability [1,2,3].

Due to the continuous development of PCM research, their range of applications has widened: PCMs are now integrated into automotive applications [4], energy storage applications [5], and heat exchangers [6]. Allouhi et al. [7] employed PCMs to optimize an energy storage system for solar water heaters operated by households in rural regions to fulfil their hot water needs at night. Huang et al. [8] used a dual-phase-change material to design a heat sink to minimize its cost and weight. Carmona et al. [9] used a PCM to improve the energy efficiency of a water storage tank for domestic applications. Their numerical model was validated with an experimental study. They found that including 40% of PCM inside the water storage tank increased its efficiency by 16%. PCMs are also used to conserve energy in the building sector as they are integrated into walls [10] and roofs [11], and can be used in indoor applications such as house furniture or decorations [12] to maintain a favorable room temperature. They can also be used in building envelopes as PCM-to-air heat exchangers where air discharges and charges the PCM with energy [13,14]. Padala et al. [15] explored the use of a PCM in a mixture for masonry blocks to determine the ideal mix proportions to attain the best mechanical, durability-related, and thermal characteristics of these mixes. Chen et al. [16] investigated the thermal performance of a thermo-activated PCM mixture integrated into a wall for energy saving purposes during winter. Gholamibozanjani et al. [17] experimentally examined incorporating PCMs to conserve energy inside huts over the seasons of a year. The results proved that the PCM storage units were able to reduce the heating/cooling energy requirements: the accumulative energy-saving varied between 10% and 40% during the year. Tyagi et al. [18] evaluated the performance of a PCM-based energy storage unit comprising panels charged by sunlight during the daytime then used during the night as a heat source for a test room. Violidakis et al. [19] discussed the use of an ultra-high-temperature PCM such as silicon in residential buildings. They possess excellent thermal conductivity and high latent heat thermal energy, achieving greater energy density and capacity. Frazzica et al. [20] produced and characterized mortar PCMs, then they introduced a new experimental setup to evaluate its thermal performance. However, PCMs still face challenges such as long discharging and changing times and low heat transfer rate due to their low thermal conductivity [21]. Thus, new technologies must be developed to improve their thermal properties and heat transfer performance. The most intuitive solution to enhance a PCM’s melting process is to extend the contact surface. Dmitruk et al. [22] studied a cylindrical PCM-based heat storage system equipped with a pin-fin structure to improve the heat transfer rate within this system. After multiple charging/discharging cycles, the experimental and numerical results showed the positive effect of the pin-fin structure on PCM performance. Sathe et al. [23] numerically analyzed the thermal-hydraulic performance a PCM melting inside a tilted finned container with a top heating mode. They observed that decreasing the inclination angles and extending the surface and fins increased the melting time for all the PCMs. Nie et al. [24] studied the thermal performance of a PCM as part of a composite containing fumed silica and graphene that was used to enhance the capability of a portable box to maintain cold temperature. According to their results, adding 1 wt% of graphene and 4 wt% of fumed silica enhanced the thermal conductivity of the PCM composite by 55.4% and helped to eliminate the PCM leakage problem. Izgi et al. [25] studied the solidification process of a PCM inside a three-dimensional cylinder. They focused on finding controlling parameters for this phenomenon. From the results, they observed that the diameter of the cylinder influenced the energy discharge and the solidification times. Sweidan et al. [26] performed multiple numerical computations to investigate the effectiveness of various techniques (multiple PCM layers, mingle PCM with highly conductive fins, and PCM-saturated metal foam) in improving PCM performance. Tarigond et al. [27] used iron scrap additives to boost the thermal performance of the PCM inside a thermal energy storage system for hot water. The results demonstrated the positive impact of these additives on the performance of the PCM, as the yield of hot water was improved by 25% compared to the control system. Ahmed et al. [28] proposed a novel design for a cascaded-layered PCM as a cost effective solution for medium-temperature industrial applications. From the results, they indicated the best volume fraction arrangement for thermal energy storage. Selimefendigil et al. [29] used FEM to evaluate the free convection of a copper oxide/water nano-liquid within a square cavity with PCM attached to its vertical wall. They found that by increasing the height of the PCM from 0.2 to 0.8 H, both the local and average Nusselt numbers decreased by 42.14%. Abu-Hamdeh et al. [30] performed a three-dimensional examination of the paraffin wax melting process in an ellipsoidal pipe with a hotter inner pipe. They discovered that the location of the inner pipe and the temperature differential influence the time required for the PCM to melt.

Nowadays, the most effective approaches for improving the thermal performance of PCMs are using a porous medium or metal foam as a support matrix for the PCM and adding high thermal conductivity nanoparticles to the PCM [31]. However, the simplest the most used method involves embedding fins in the PCM containers. Over the years, several studies have been conducted on the influence of fins on heat transfer in various media, including PCMs [32,33,34,35].

Jeong et al. [36] reported that the thermal properties of three different shape-stabilized PCM composites were enhanced by adding exfoliated graphite nanoplatelets (xGnP). Nóbrega et al. [37] investigated the influence of adding fins to a tube filled with a nano-PCM mixture. The findings suggested that the fins helped decrease the solidification time by 9.1%, and increasing the concentration of nanoparticles improved the solidification process regardless of the presence of fins. Raj et al. [38] packed a nano-enriched PCM inside a wall-less heat sink used in a thermal management application. The study results revealed that the addition of MWCNTs and GnP nanoparticles to the FS-PCM enhanced its thermal conductivity by 61.73% and 84.48%, respectively, thus improving the heat transfer performance. Jourabian et al. [39] investigated the melting behavior of ice as a PCM within a horizontal elliptical tube filled with nickel-steel porous media. Das et al. [40] addressed the form stability of a PCM subjected to numerous cycles of charging and discharging by producing a novel biocomposite-based PCM. The testing results indicated that using biochar from *Eichornia crassipes* as a supporting matrix mix with a small amount of aluminum metal powder enhanced the thermal conductivity of the PCM 17.27 times and improved its overall stability. Vennapusa et al. [41] tested six lightweight support materials to improve the form stability of a caprylic-acid-based PCM. From the results, they concluded that using expanded perlite as a support matrix for the PCM achieved the highest enthalpy and thermal buffering.

Rathore et al. [42] added expanded vermiculite and expanded graphite to improve the thermophysical properties of a low-cost PCM. The study results revealed that the thermal conductivity increased by 114.4% when the PCM was loaded with 7% of EG. Moreover, the enhanced PCM preserved its thermal properties even after 1000 charging and discharging cycles. Qureshi et al. [43] employed TPMS-based metal foams with a 3D-printed structures as a skeleton for MFPCMs to improve the thermal conductivity of traditional PCMs. According to their results, the thermal conductivity of MFPCMs was strongly affected by the cell type and its unique shape, in addition to the cell porosity. Combining the two approaches, Nada et al. [44] discussed enhancing paraffin wax as a PCM in an energy storage unit by using a carbon foam matrix and MWCNTs additives. Mehryan et al. [45] studied the non-Newtonian behavior in space between two coaxial pipes filled with metal foam. They reported that a 54% reduction in the melting time could be achieved by lowering the power law index from 1 to 0.6.

The literature review revealed that entropy formation during the charging process has been not well-investigated. As a result, in this study, we used GFEM to replicate NEPCM’s second-law behavior during melting. The contours of entropy component, temperature, and melt fraction are shown.

## 2. Methodology and Problem Definition

**Figure 1:**
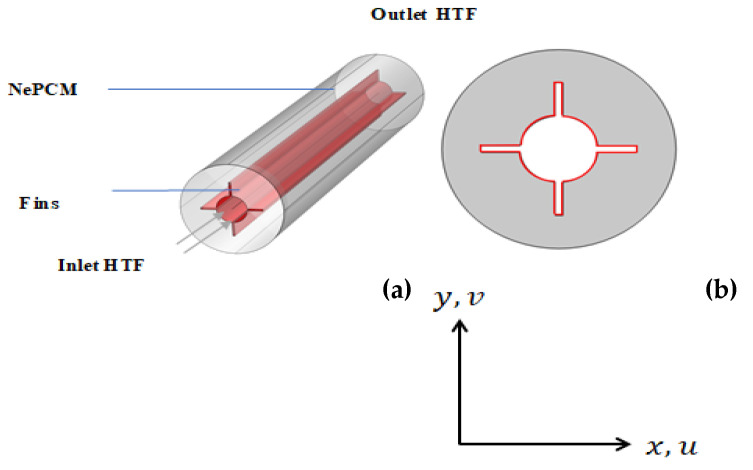
Computational setup of LTESS: (**a**) 3D model (**b**) 2D cross-section.

**Figure 2:**
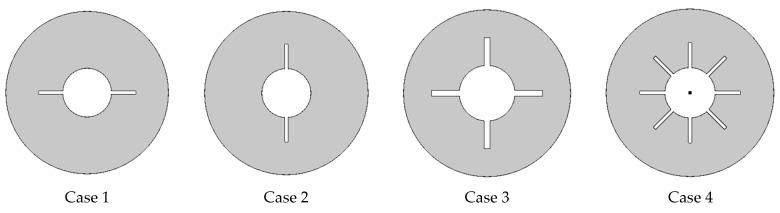
Different cases considered in this study.

Figure 1 depicts an illustration of the current computational models. Al_2_O_3_/n-octadecane paraffin was used as the working fluid. Fins were modeled in four different configurations. Figure 2 depicts the specifics of four examples. At the start, all fluids had a solidus temperature. The fins and inner cylinder are maintained at hot temperature (312 K), and the outer cylinder is adiabatic.

### 2.1. Problem Formulation

To model this transitory process, we assumed that the flow was Newtonian and laminar. To account for the gravity force effect, Boussinesq estimation was used. The calculation formulas are as follows [46,47,48,49]:

(1) $$\nabla \cdot \vec{V}=0$$

(2) $$\left(\frac{\partial v}{\partial t}+\vec{V}\cdot \nabla v\right)=vC\frac{{\left(\lambda -1\right)}^{2}}{\varepsilon +\lambda^{3}}+\frac{1}{\rho_{nf}}\left(-\nabla P+\mu_{nf}\nabla^{2}v\right)+\frac{1}{\rho_{nf}}{\left(\rho \beta \right)}_{nf}g\left(T-T_{ref}\right)$$

(3) $$\frac{\partial u}{\partial t}+\vec{V}\cdot \nabla u=uC\frac{{\left(\lambda -1\right)}^{2}}{\varepsilon +\lambda^{3}}+\frac{1}{\rho_{nf}}\left(-\nabla P+\mu_{nf}\nabla^{2}u\right)$$

(4) $${\left(\rho C_{p}\right)}_{nf}\frac{\partial {\left(\rho L\lambda \right)}_{nf}}{\partial t}+{\left(\rho C_{p}\right)}_{nf}\frac{\partial T}{\partial t}-k_{nf}\nabla^{2}T=-{\left(\rho C_{p}\right)}_{nf}\vec{V}\cdot \nabla T$$

We considered $\varepsilon =10^{-3}\;\mathrm{and}\;C=10^{5}$.

To forecast the NEPCM attributes, a single-phase model was used:

(5) $${\left(\rho C_{p}\right)}_{f}^{-1}{\left(\rho C_{p}\right)}_{nf}=\left(1-\phi \right)+\phi {\left(\rho C_{p}\right)}_{s}{\left(\rho C_{p}\right)}_{f}^{-1}\;$$

(6) $$\rho_{nf}=\phi \rho_{s}+\rho_{f}\left(1-\phi \right)$$

(7) $${\left(\rho \beta \right)}_{nf}=\phi {\left(\rho \beta \right)}_{s}+\left(1-\phi \right){\left(\rho \beta \right)}_{f}$$

(8) $${\left(\rho L\right)}_{f}=\frac{{\left(\rho L\right)}_{nf}}{\left(1-\phi \right)}$$

(9) $$k_{nf}=\frac{2k_{f}+2\phi \left(k_{s}-k_{f}\right)+k_{p}}{k_{p}-\phi \left(k_{s}-k_{f}\right)+2k_{f}}k_{f}$$

(10) $$\mu_{nf}=\frac{\mu_{f}}{{\left(1-\phi \right)}^{2.5}}$$

Table 1 lists the characteristics of both nanoparticles and the PCM.

The enthalpy is formulated as [50]:

(11) $$h=h_{ref}+\int_{T_{\mathrm{ret}\;}}^{T}{\left(C_{p}\right)}_{nf}dT$$

(12) $$\lambda =\left\{\begin{array}{cc} 1 & T<T_{l}\\ \frac{T-T_{s}}{T_{l}-T_{s}} & T_{s}<T<T_{l},H_{e}=h+\lambda L\\ 0 & T<T_{s} \end{array}\right.$$

The formulas of $S_{\mathrm{gen},\;\mathrm{total}\;},S_{\mathrm{gen},\;\mathrm{th}\;},\mathrm{and}\;S_{\mathrm{gen},\;f\;}$ are:

(13) $$\begin{array}{ll} S_{\mathrm{gen},\mathrm{total}\;}= & S_{\mathrm{gen},\mathrm{th}\;}+S_{\mathrm{gen},f}\\ = & \frac{k_{nf}}{T^{2}}\left[{\left(\frac{\partial T}{\partial x}\right)}^{2}+{\left(\frac{\partial T}{\partial y}\right)}^{2}\right]\\ & +\frac{\mu_{nf}}{T}\left\{2\left[{\left(\frac{\partial u_{x}}{\partial x}\right)}^{2}+{\left(\frac{\partial u_{y}}{\partial y}\right)}^{2}\right]+{\left(\frac{\partial u_{x}}{\partial y}+\frac{\partial u_{y}}{\partial x}\right)}^{2}\right\} \end{array}$$

The no-slip boundary conditions are subjected to the previous system (u=v=T=0) on the outer boundaries, while on the inner fins $u=v=0,\;T=T_{h}.$

### 2.2. GFEM Approach

**Figure 3:**
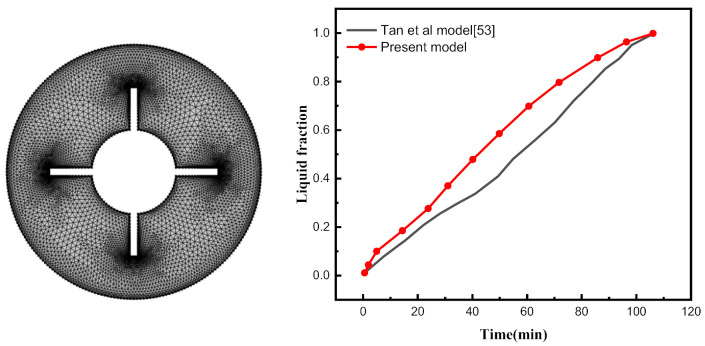
Mesh grid and of the present model in comparison to [53].

The Galerkin finite element method handles the transformed coupled Equations (1)–(4), which include both the flow and heat transfer phenomena as well as the aforementioned boundary conditions. The governing equations’ weak forms are given and discretized on a nonuniform structural grid. The results are then simulated using mathematical software. The process is described in full in [51,52]. The validation of the present code was obtained and is displayed in Figure 3 using the additional numerical results of Tan et al. [53]. We are confident in our results based on this figure.

## 3. Results and Discussion

In this section, we illustrate and interpret the results obtained from examining the melting effects on the flow of a suspension that contains the phase change material (PCM). Here, the worked fluid was Al_2_O_3_/n-octadecane paraffin and the flow area was a cylinder tube including cross-section fins. Features of the temperature, velocity, Bejan number, and liquid fraction were examined for various heating cases, namely, a cylinder with two horizontal wings, a cylinder with two vertical wings, a cylinder with four wings, and a cylinder with eight heated fins. The variations in the time of required ranged from 100 and 600 s and the values of the nanoparticles volume fraction were considered between φ=0% and 8%. To present a comprehensive investigation, the average values of the liquid fraction β, Bejan number $Be_{avg}$, and the rate of the heat transfer $Nu_{avg}$ over time are presented graphically for a wide range of the considered parameters. Additionally, the condition of the totally melted NEPCM (liquid fraction = 1) may be used to terminate the computations.

**Figure 4:**
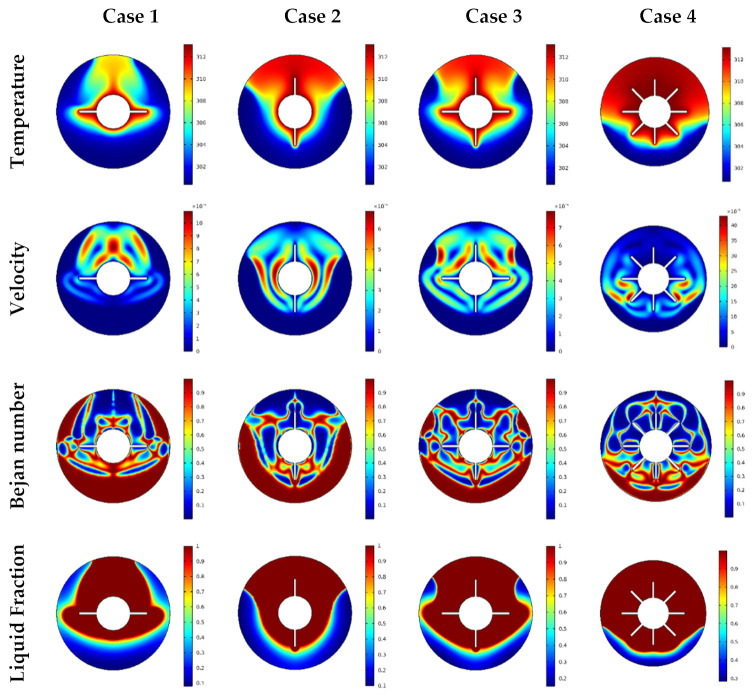
Temperature, velocity, Bejan number, and liquid fraction contour in different geometries.

Figure 4 displays the temperature, velocity, local Bejan number, and the liquid fraction for various cases of inner heating. Notably, the temperature features concentrated around the fins in all the cases, with a cold zone indicated near the bottom of the outer cylinder. These temperature distributions achieved their maximum values in case 4 (eight wings) pointing to a decrease in the aforementioned cold zone at the bottom. We also observed that the increase in number of wings reduced the temperature differences; hence, both the temperature gradients and heat transfer rate diminished. Additionally, a clear reduction in the velocity values was noted as the number of wings increased. Physically, the increase in the number of wings enhances the complexity in the flow area; hence, the flow resistance is augmented. In the same context, the features of the Bejan number showed that the increase in the number of heated fins reduced the gradients of the temperature; hence, the fluid friction irreversibility became dominant. Furthermore, the melting zone was seen in the upper half of the domain for all the considered cases as the increase in number of heated fins enhanced the melted zone.

**Figure 5:**
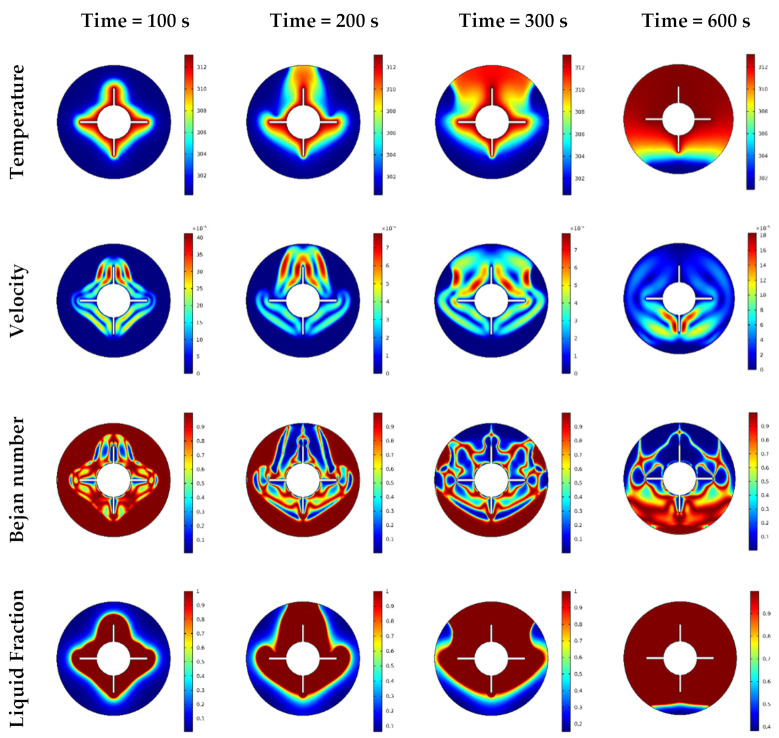
Temperature, velocity, Bejan number, and liquid fraction contour in different time steps.

The features of the temperature, velocity, local Be number, and local liquid fraction with variations in time are depicted in Figure 5. During these computations, case 3 used an inner cylinder with four wings. The results indicated that at the beginning of the calculations (small values of the time), the distributions of the temperature, velocity, and Bejan number occurred around the inner part heated, indicating a nonactive zone near the outer boundaries. Over time, the fluid started to carry and distribute the temperature throughout the whole domain. Therefore at *t* =600 s, a good thermal domain was obtained with a higher velocity rate near the bottom of the outer boundaries. Additionally, for higher time values, the fluid friction irreversibility near the bottom dominated compared to the heat transfer irreversibility. From the physical viewpoint, this behavior is due to the gradients of the velocity that enhance with time, resulting in an augmentation in fluid friction irreversibility. Furthermore, a mushy zone was observed within the full flow domain with increasing time.

**Figure 6:**
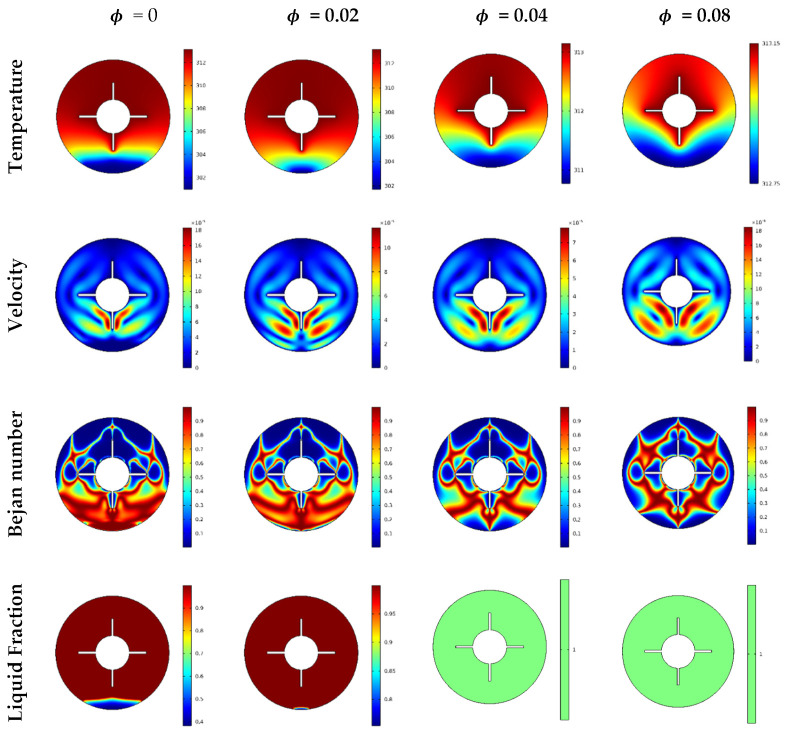
Temperature, velocity, Bejan number, and liquid fraction contour of nanoparticles’ concentrations.

Figure 6 shows the distributions of the temperature, velocity, local Bejan number, and liquid fraction under impacts of the volume fraction parameter φ. The inner heated cylinders with four wings were used in this case. We noted a low convective transport at higher values of φ due to the increase in the viscosity of the mixture. The results indicated diminishing velocity and temperature gradients with increasing φ. Additionally, the local Bejan number occurred around the wings instead of the bottom boundaries at low values of φ. Conversely, the increase in φ enhanced the mushy zone within the flow area until completely melted conditions were obtained at φ≥0.04.

**Figure 7:**
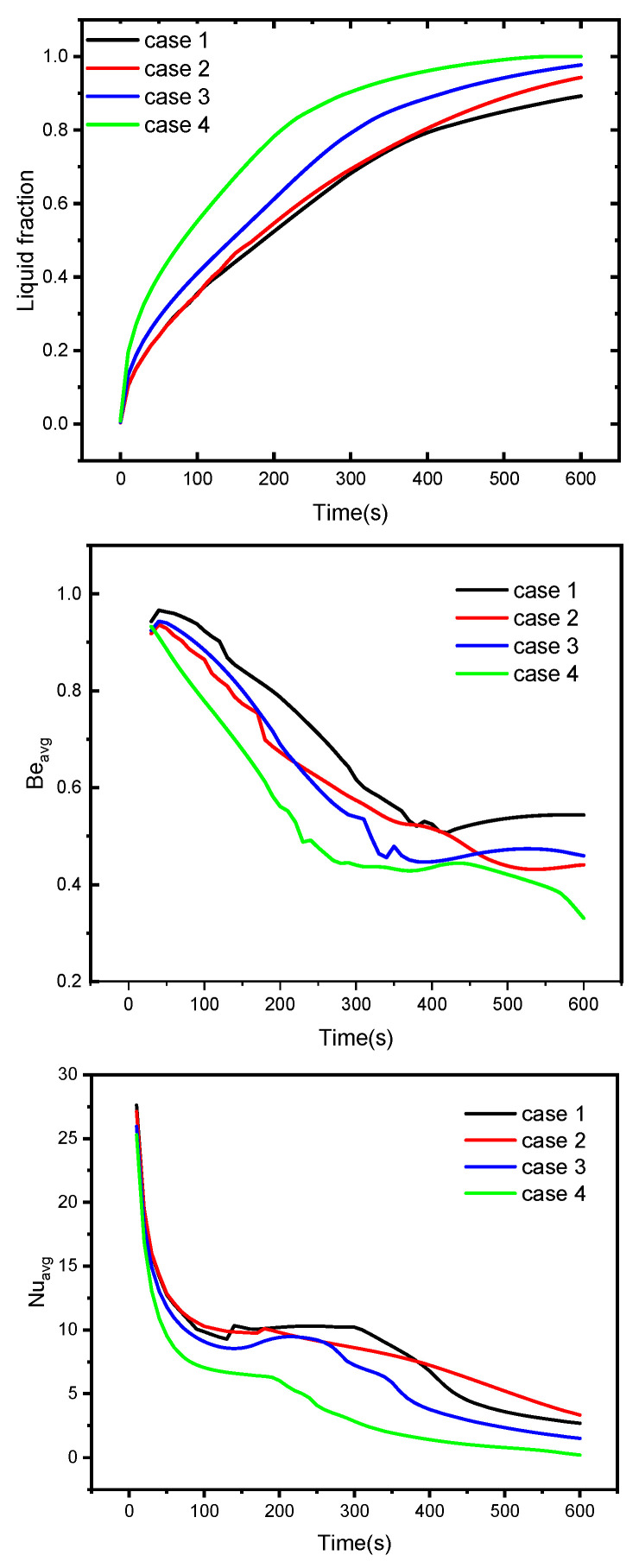
The influences of geometry on the liquid fraction, Nusselt number, and Bejan number.

**Figure 8:**
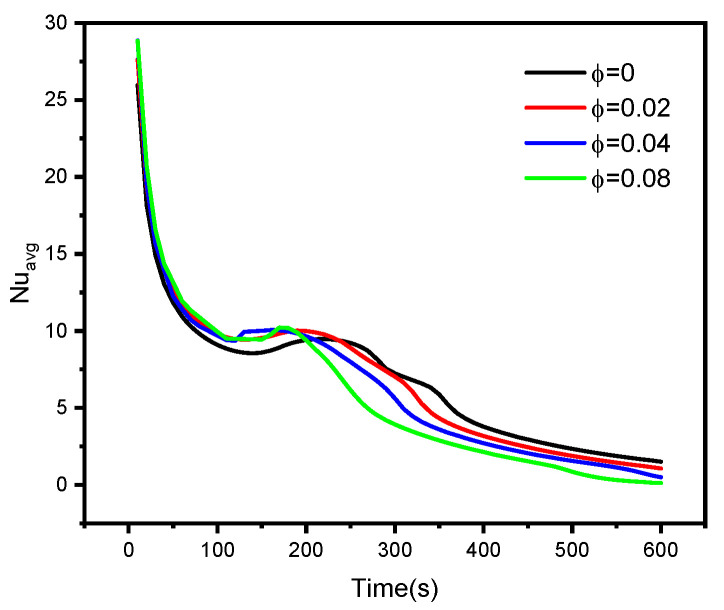

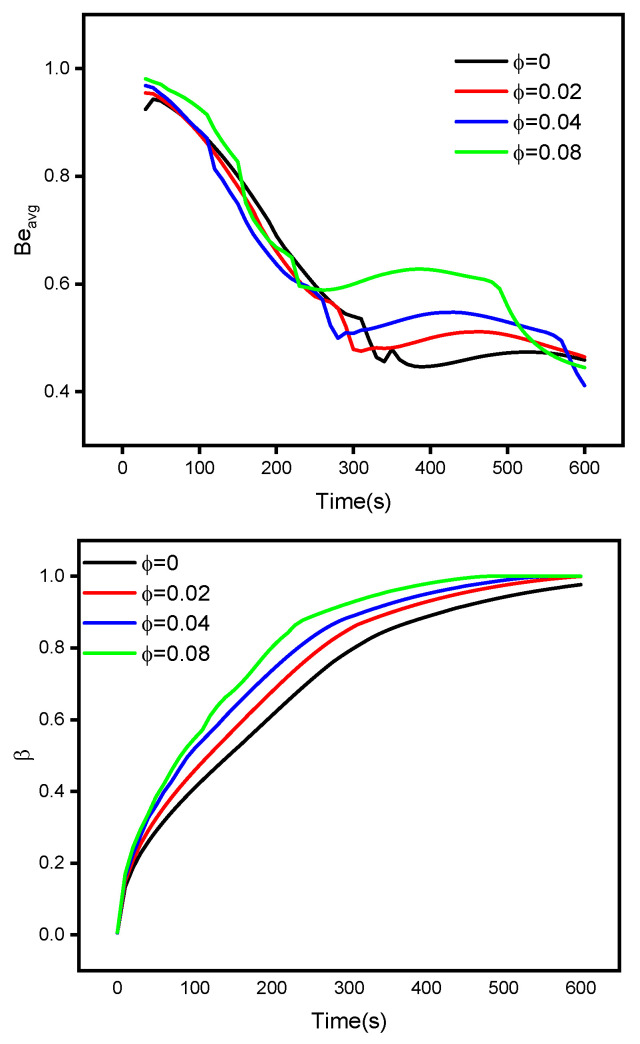
The influences of nanoparticles’ concentration on liquid fraction, Nusselt. Number, and Bejan number.

Figure 7 and Figure 8 illustrate the profiles of the average liquid fraction β, average Bejan number $Be_{avg},\;$ and average Nusselt number $Nu_{avg}$ under with different numbers of heated wings, time parameters, and volume fraction parameters. The results revealed that case 4, in which eight heated wings were assumed, produced the highest average liquid fraction values. However, the average Bejan and Nusselt numbers decreased as the number of heated wings increased. Additionally, the average rate of heat transfer diminished as φ increased due to the decrease in the temperature gradients. Furthermore, higher values of φ caused the irreversibility of the heat transfer to be dominant compared to the fluid friction irreversibility. Finally, we observed that the increase in the volume fraction parameter φ enhanced the mushy zone and, hence, the average liquid fraction rose.

## 4. Conclusions

This paper presented a numerical investigation into the impacts of melting on the convective flow of phase change materials within cylindrical tubes containing cross-shape heated sections. Four cases were considered based on the number of heated wings, namely, case 1 (two horizontal wings), case 2 (two vertical wings), case 3 (four wings), and case 4 (eight heated wings). The unsteady case was considered and completely melted conditions were assumed. The finite element method (FEM) with the Poisson pressure equation was applied to solve the governing system. The following are our major findings:

- Distributions of the temperature, velocity, and Bejan number increase as with an increasing number of heated wings due to the augmentation in the buoyancy convective case. Additionally, the melted area was controlled for the most of the flow domain in case 4.
- For small time values, the increases in temperature, velocity, and liquid fraction occur around the inner heated shapes, but over time, a good isothermal and melted flow domain is obtained.
- Increases in φ cause an enhancement in the dynamic viscosity of the mixture; hence, the velocity decreases as φ increases.
- With time, the irreversibility due to fluid friction becomes more dominant compare to heat transfer irreversibility.

## Figures and Tables

**Table 1:** Properties of the PCM and alumina [33].

Property	Al_2_O_3_	n-octadecane
ρ (kg/m^3^)	3970	770
$\beta \times 10^{5}\;$(K^–1^)	0.85	91
k (w/mK)	40	0.157
L (j/kg)	—	242.9
$\mathrm{Fusion}\;\left(C\right)$	—	28 × 10^3^
$\mu \times 10^{3}$ (Pa s)	—	3.79
$C_{p}$ (j/kg K)	765	2189

## Data Availability

All data are available upon request from any of the authors.

## Nomenclature

g	Gravity
$T_{m}$	Fusion temperature
C	Mushy zone constant
NEPCM	Nanoenhanced PCM
$L_{f}$	Latent heat coefficient
k	Thermal conductivity
$T_{s}$	Solidus temperature
FVM	Finite volume method
$T_{l}$	Liquidus temperature
**Greek symbols**	
α	Thermal diffusivity (m^2^/s)
ρ	Fluid density
ϕ	Nanoparticle volume fraction
**Subscripts**	
nf	NEPCM
f	Pure fluid
